# Impacts of climate change and human activities on vegetation coverage variation in mountainous and hilly areas in Central South of Shandong Province based on tree-ring

**DOI:** 10.3389/fpls.2023.1158221

**Published:** 2023-06-05

**Authors:** Tingting Yin, Yinuo Zhai, Yan Zhang, Wenjun Yang, Jinbin Dong, Xiao Liu, Peixian Fan, Chao You, Linqian Yu, Qun Gao, Hui Wang, Peiming Zheng, Renqing Wang

**Affiliations:** ^1^ Institute of Ecology and Biodiversity, School of Life Sciences, Shandong University, Qingdao, China; ^2^ Shandong Provincial Engineering and Technology Research Center for Vegetation Ecology, Shandong University, Qingdao, China; ^3^ Qingdao Forest Ecology Research Station of National Forestry and Grassland Administration, Shandong University, Qingdao, China; ^4^ Shandong Huankeyuan Environmental Testing Co., Ltd., Jinan, China

**Keywords:** anthropogenic activities, central-south shandong, climatic variation, dendrochronology, vegetation cover dynamic

## Abstract

**Introduction:**

It is of great significance to understand the characteristics and influencing factors of vegetation coverage variation in the warm temperate zone. As a typical region of the warm temperate zone in eastern China, the mountainous and hilly region in central-south Shandong Province has fragile ecological environment and soil erosion problem. Studying on vegetation dynamics and its influencing factors in this region will help to better understand the relationship between climate change and vegetation cover change in the warm temperate zone of eastern China, and the influence of human activities on vegetation cover dynamics.

**Methods:**

Based on dendrochronology, a standard tree-ring width chronology was established in the mountainous and hilly region of central-south Shandong Province, and the vegetation coverage from 1905 to 2020 was reconstructed to reveal the dynamic change characteristics of vegetation cover in this region. Secondly, the influence of climate factors and human activities on the dynamic change of vegetation cover was discussed through correlation analysis and residual analysis.

**Results and discussion:**

In the reconstructed sequence, 23 years had high vegetation coverage and 15 years had low vegetation coverage. After low-pass filtering, the vegetation coverage of 1911–1913, 1945–1951, 1958–1962, 1994–1996, and 2007–2011 was relatively high, while the vegetation coverage of 1925–1927, 1936–1942, 2001–2003, and 2019–2020 was relatively low. Although precipitation determined the variation of vegetation coverage in this study area, the impacts of human activities on the change of vegetation coverage in the past decades cannot be ignored. With the development of social economy and the acceleration of urbanization, the vegetation coverage declined. Since the beginning of the 21st century, ecological projects such as Grain-for-Green have increased the vegetation coverage.

## 1 Introduction

Vegetation is an important part of the terrestrial ecosystem; It not only connects the lithosphere, atmosphere, and hydrosphere, but also plays a crucial role in maintaining the global ecological balance (Guo et al., 2021). Vegetation variation is often seen as an indicator of environmental change at various spatial and temporal scales (Wen et al., 2017; Qi et al., 2019) and is thought to be triggered by climatic factors, human activities, or both (Rong et al., 2016). In recent years, due to climate change and different levels of interference from human activities, vegetation has been degraded or restored to different degrees, affecting biodiversity to a certain extent.

In the past few decades, the global climate has undergone several unprecedented changes, and global warming has become an indisputable fact (Gao et al., 2022). A series of events triggered by climate change, such as temperature changes, precipitation fluctuations, and radiation changes, have led to dramatic changes in vegetation cover in many parts of the globe (Liu et al., 2021a; IPCC, 2022). Significant temperature increases lead to increased evapotranspiration, which may negatively affect vegetation growth, while the change of precipitation distribution pattern may lead to different degrees of vegetation degradation or increase in different areas (Liu et al., 2021b; Yang et al., 2022). In addition, human activities have clearly influenced the change in vegetation cover (Gao et al., 2022; Song et al., 2022). Human beings make policies and implement ecological projects such as Grain-for-Green for closing the mountain for reforestation or precise improvement project of forest quality (Liu et al., 2023) to increase vegetation coverage (Wen et al., 2017; Zhang et al., 2022), while factors such as rapid population growth, accelerated urbanization process, unreasonable reclamation and increase of cultivated land area will reduce vegetation coverage (Dong et al., 2020; Gao et al., 2022).

Normalized vegetation index (NDVI) can accurately reflect important information such as vegetation green dynamics, productivity, biomass, vegetation cover, and growth status, so it has been widely used to study vegetation dynamic change and its response mechanism (Hossain and Li, 2021; Gu et al., 2022). However, because of its short time scale, it cannot reflect the vegetation change at a longer time scale. Therefore, it is crucial to find reliable substitutes for vegetation change. Tree rings are widely used in the study of past climatic, ecological, and environmental changes due to their accurate dating, strong continuity, high resolution, and sensitivity to the environment (Zhang, 2015). Since the radial growth of trees and vegetation growth in the same area are affected by common climatic and environmental factors (Xu et al., 2021), the use of tree rings to study the change of vegetation cover can make up for the short time scale of NDVI remote sensing data.

At present, research using tree rings in China mainly focused on climate reconstruction in extreme climate zones such as alpine or arid regions (Guang et al., 2015; Xiao et al., 2021), and runoff reconstruction (Li et al., 2018; Zhang et al., 2020; Gao et al., 2021). And the study of vegetation change using tree rings is mainly concentrated in northwest, northeast, and subtropical regions of China (Li et al., 2021; Dong et al., 2022), while few studied in eastern China. Shandong province is located in the northern hemisphere mid-latitudes, squarely in the north and the south of the transition zone, and is located in the warm temperate zone of the east Asia monsoon region (Wang et al., 2013; Guo et al., 2017). The developed soil texture is mainly sandy, loam, clay and gravel in the mountainous and hilly area of central-south Shandong, and the soil types are mainly brown soil, brown soil, tidal soil, mortar black soil and coarse bone soil (Zhai, 2018; Yan, 2021). *Platycladus orientalis* (L.) Franco is considered as a good tree species for dendrology because of its long lifespan, strong adaptability, sensitivity to climate change, and clear ring boundaries. The mountainous and hilly areas in the central south of Shandong Province as an important geographical unit is a subtropical and temperate climate transition zone and climate-sensitive area with fragile ecological environment and serious problem of soil and water loss (Yan, 2021). Therefore, it is of great practical significance to study the various characteristics and influencing factors of vegetation coverage in the mountainous and hilly areas of central-south Shandong Province.

By analyzing the relationship among climate factors, tree rings, and NDVI, this study reconstructed the vegetation coverage in the mountainous and hilly area of central-south Shandong Province since 1905, and analyzed the impacts of climate factors and human activities on vegetation coverage change to provide some basic data for the historical vegetation cover change under the warm temperate monsoon climate.

## 2 Materials and methods

### 2.1 Study area

**Figure 1:**
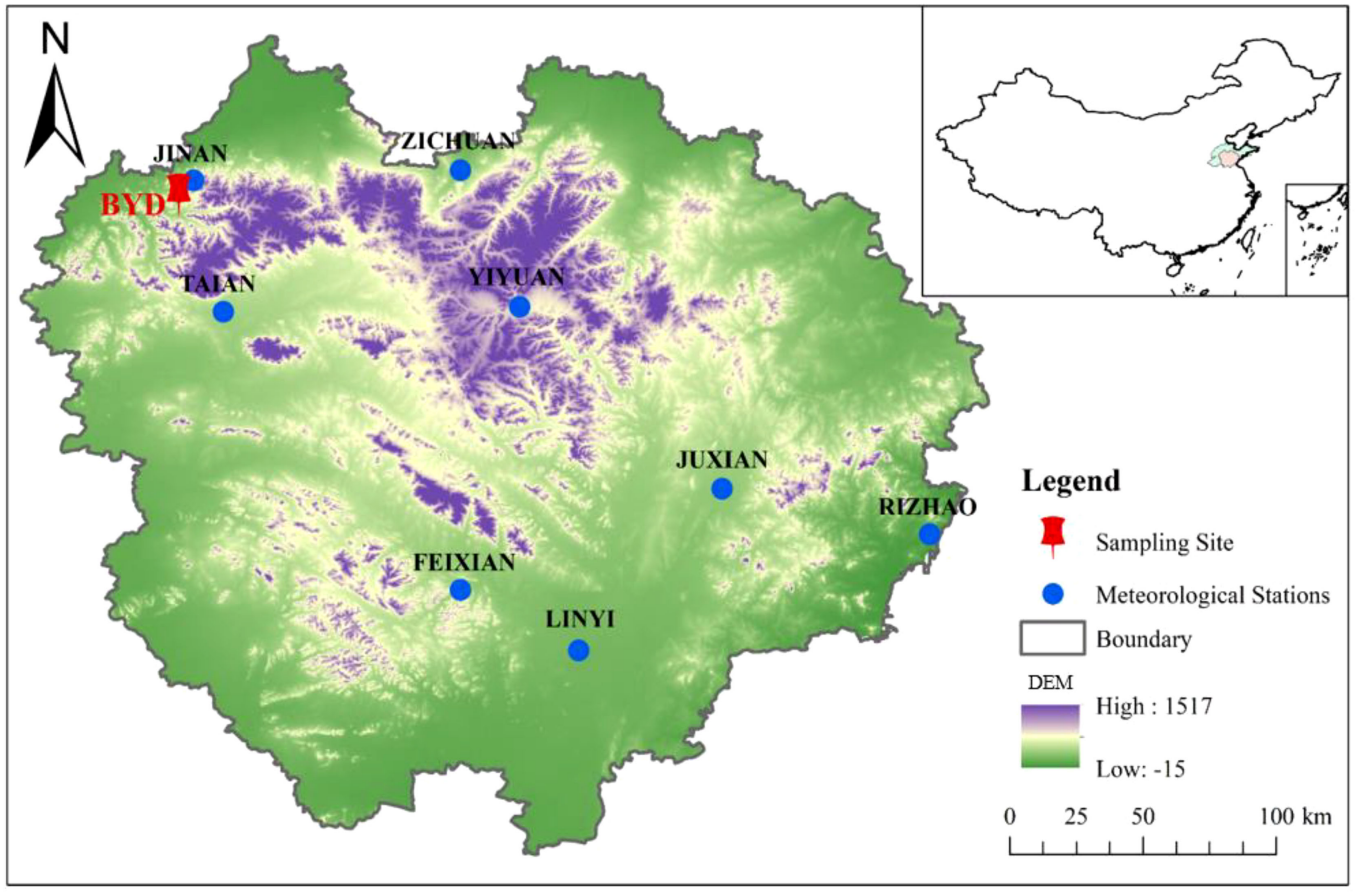
Geographical location of study sites and meteorological stations. (DEM, Digital Elevation Model).

**Figure 2:**
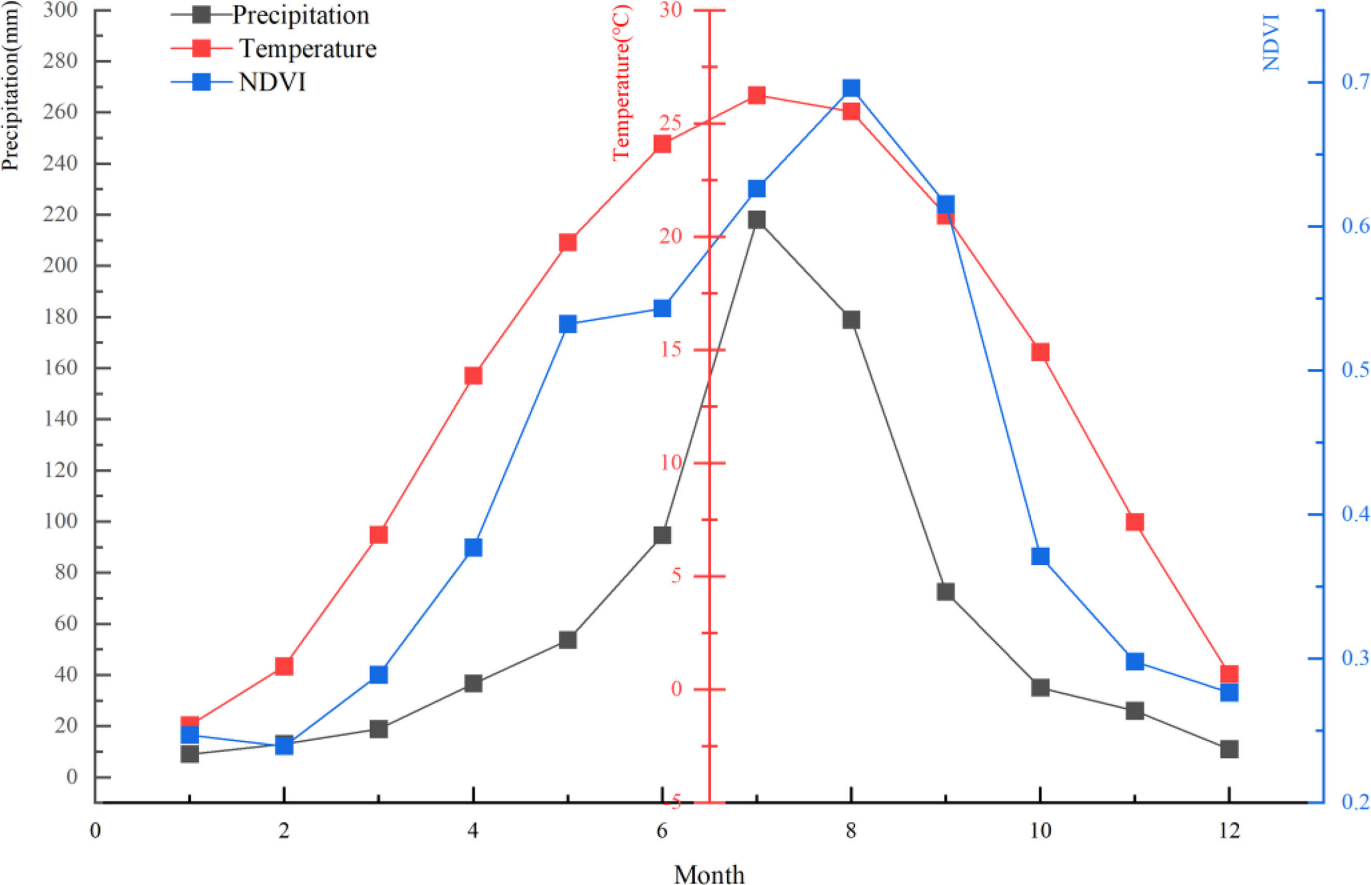
Climate information of the study area. (The red curve represents average monthly temperature, the blue curve represents average monthly NDVI, and the black curve represents average monthly precipitation).

The study area was located in central-south Shandong Province as an important geographical unit of Shandong Province (**Figure 1**), and was the central area with the most prominent topographic elevation in Shandong almost surrounded by alluvial plains. The mean annual temperature in this region is –1.56–26.25°C, and the mean annual precipitation is 212.8 mm (**Figure 2**). The climate type is warm temperate monsoon climate, with four distinct seasons, cold and dry in winter, hot and rainy in summer, and hot and rainy in the same period (Liu et al., 2020).

The vegetation types in this area are rich, mostly in temperate deciduous broad-leaved forest, and the vegetation growing season is from May to September. *P. orientalis* is the dominant species in more than 80% of the sampling sites. However, because of the influence of climate, topography, humans, and other factors, the vegetation in this area has been seriously damaged.

### 2.2 Dataset

#### 2.2.1 Tree-ring data

The samples were collected from *P. orientalis* at Dingjiayu in the southern mountain area of Jinan, Shandong Province. 156 tree cores were drilled using increment borers according to standard procedures defined by the International Tree Dendrochronology Database (ITRDB) (Cook and Kairiukstis, 1990; Peter, 2014). The tree ring width was measured using LINTAB 6 (0.001 mm) after air drying, fixing, and polishing. COFECHA was cross-checked for dating (Gao et al., 2022; Song et al., 2022). Using COFECHA to compare the statistical correlation of tree ring sequences, and the possible problem tree-rings were observed under the microscope to find out the problem and correct it. Samples that are broken or grown too peculiar to accurately cross-date will be eliminated. The mean sensitivity of the sequences in COFECHA was 0.477, and the series intercorrelation was 0.390.

**Figure 3:**
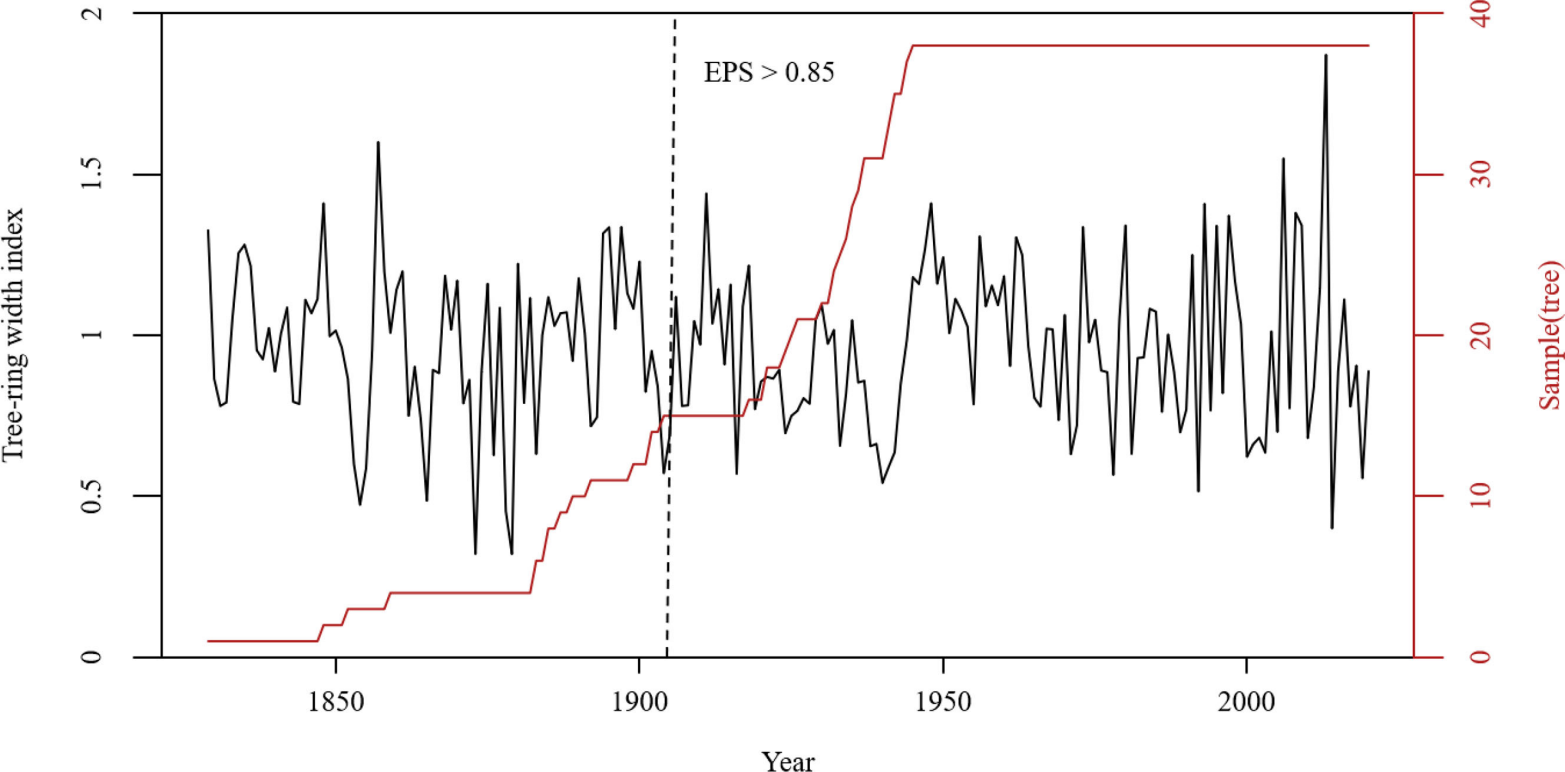
The regional tree-ring standard chronology. The reliable portion of the chronology is determined by the EPS value being > 0.85. (Expressed Population Signal, EPS: The representation of the sample to the population).

And then the standard chronology was developed using ARSTAN (Cook and Kairiukstis, 1990). Negative exponential or linear functions were used to remove tree growth trends. After excluding cores with worse correlations with the master series, a regional chronology (**Figure 3**) was developed. The chronology spans the period from 1829 to 2020, and the reliable interval is 1905–2020 (EPS > 0.85). The statistical parameters of standard chronology are shown in **Table 1**.

**Table 1:** Robust mean standard chronology statistics.

Sampling Sites	Time Span (Year)	Mean Sensitivity (MS)	Series Intercorrelation	Expressed Population Signal (EPS)	Mean Index	Standard Deviation (SD)	First year where EPS > 0.85 (Number of Trees)
DJY	1829−2020,192	0.266	0.129	0.926	0.957	0.253	1905(15)

#### 2.2.2 Climate data

Meteorological data, including monthly precipitation, monthly mean temperature, and relative humidity were obtained from the National Weather Science Data Center (http://data.cma.cn/). And the time span of them is 1952–2020. To reduce small-scale noise or random components and enable meteorological data to be representative of a wider range of regional climate conditions, we used arithmetic mean datasets from multiple weather stations for further analysis.

#### 2.2.3 NDVI data

The NDVI dataset used in this study is the global GIMMS NDVI3g v1 dataset (1981–2015) from http://data.tpdc.ac.cn/en/data/ that has been corrected for calibration, view geometry, volcanic aerosols, and other effects not related to vegetation change. The period of the product is from July 1981 to December 2015 with a twice-monthly temporal resolution and 1/12° of a degree for spatial resolution. As one of the most widely used vegetation indices, NDVI can better reflect vegetation cover, productivity, and biomass, so we used the obtained NDVI to represent regional vegetation cover change.

### 2.3 Methods

A maximum value composite (MVC) method was used to calculate the monthly average NDVI value of the study area (Chu et al., 2018). To study the relationship among NDVI, tree rings, and climate factors and determine the limiting climate factors affecting NDVI and tree radial growth, Pearson correlation analysis method was used to analyze the correlation among the three factors. Then NDVI was reconstructed by linear regression model (Wen et al., 2017). To test the reliability of the reconstructed model, we used the separation calibration test. The error reduction (RE), effective coefficient (CE), sign test (ST), and first difference sign test (ST1) were calculated to verify the stability of the climate reconstruction model (Cook and Kairiukstis, 1990). The residual analysis method can strip the influence of climate factors on NDVI, so we used the residual trend method to calculate the relative contributions of climate change and human activities to vegetation cover change (Gu et al., 2022).

## 3 Results and discussion

### 3.1 Relationship among tree rings, NDVI, and climate factors

**Figure 4:**
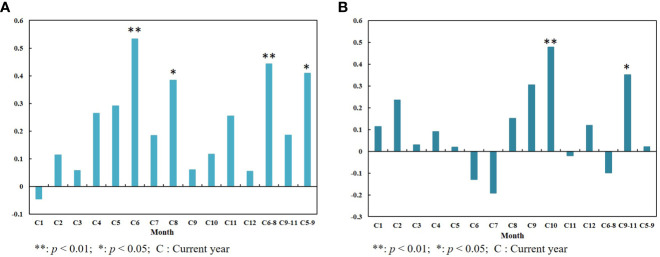
Correlation between tree-ring width index and NDVI: **(A)** NDVI and tree-ring width index of the current year; **(B)** NDVI and tree-ring width index of the next year.

By analyzing the correlation between tree rings and summer (June–August) NDVI (Vegetation coverage is higher at this time) during their common period (1982–2015), we know that there is a certain correlation between radial growth of *P. orientalis* and the NDVI. From **Figure 4**, we knew the tree-ring width of the current year was positively correlated with the NDVI in summer and the whole growing season, while autumn (September–November) NDVI had a positive effect on the radial growth of trees of the next year. The high positive correlation showed that the radial growth of trees in the study area was consistent with the trend of NDVI, which may be controlled by common climatic factors (Sun et al., 2020). NDVI mainly reflects the green degree of plant leaves and is closely related to the photosynthetic activity of vegetation (Wang et al., 2014). The width of tree rings represents the radial growth of trees, and the growth rate is mainly determined by the net accumulation of photosynthesis and respiration (Kannenberg et al., 2019). Chlorophyll is very important for plant photosynthesis, and its content will determine the accumulation of photosynthesis, and then affect the radial growth of plants (Wang et al., 2010). Therefore, when limiting factors are applied to vegetation, the photosynthesis of plants is weakened and plant growth is inhibited, thus reducing tree ring width and NDVI.

**Figure 5:**
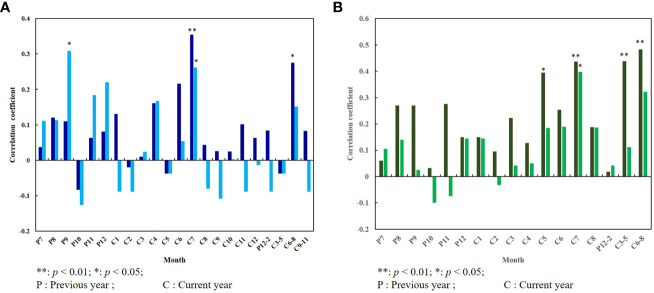
Correlation between tree-ring width index, summer NDVI and climate factors: **(A)** Tree-ring width index and mean monthly precipitation (light blue) and mean monthly relative humidity (dark blue); **(B)** Summer NDVI and mean monthly precipitation (dark green) and mean monthly relative humidity (light green).

To further demonstrate that tree rings can reflect changes in NDVI, we analyzed the responses of tree-ring width index and June–August NDVI to climate during their common periods. It was found that the radial growth of *P. orientalis* had a significant positive correlation with the monthly average precipitation in September of the previous year and the current July, and showed a significant positive correlation with the relative humidity in current July and summer by analyzing the correlation between tree-ring width and climatic factors (**Figure 5A**). This result is consistent with the results of the study on the ring climate of *Pinus thunbergii* Parlatore in Mengshan Mountain (Chen et al., 2014) and *Pinus tabuliformis* Carriere in Yishan Mountain (Xu and Zhao, 2015) of Shandong Province. According to the correlation between summer NDVI and climatic factors, the average monthly precipitation in May, July, spring (March–May), and summer of the current year had a positive impact on summer NDVI (**Figure 5B**).

These results indicated that the radial growth of *P. orientalis* and summer NDVI were limited by the same climatic factor (precipitation) in the mountainous and hilly region of central-south Shandong Province. And the radial growth of trees had a certain correlation with NDVI. In addition, trees are an important form of vegetation, and their growth state represents a partial change in vegetation coverage (Dong et al., 2022). Therefore, the tree-ring width index can reflect changes in summer vegetation cover.

### 3.2 NDVI reconstruction from 1905 to 2020

Based on the above correlation analysis, we used a linear regression model to reconstruct the summer NDVI(1905–2020). The transfer function is as follows:


, $$NDVI_{total}=0.04176STD_{t}+0.55971$$

(*n* = 34, 1982–2015 CE, *r* = 0.44, *R* ^2 = ^0.20, *R* ^2^_adj_=0.17, *p*< 0.01),

where STDt is the tree-ring index for a given year. The model explained 20% of the variance in the NDVI data.

**Figure 6:**
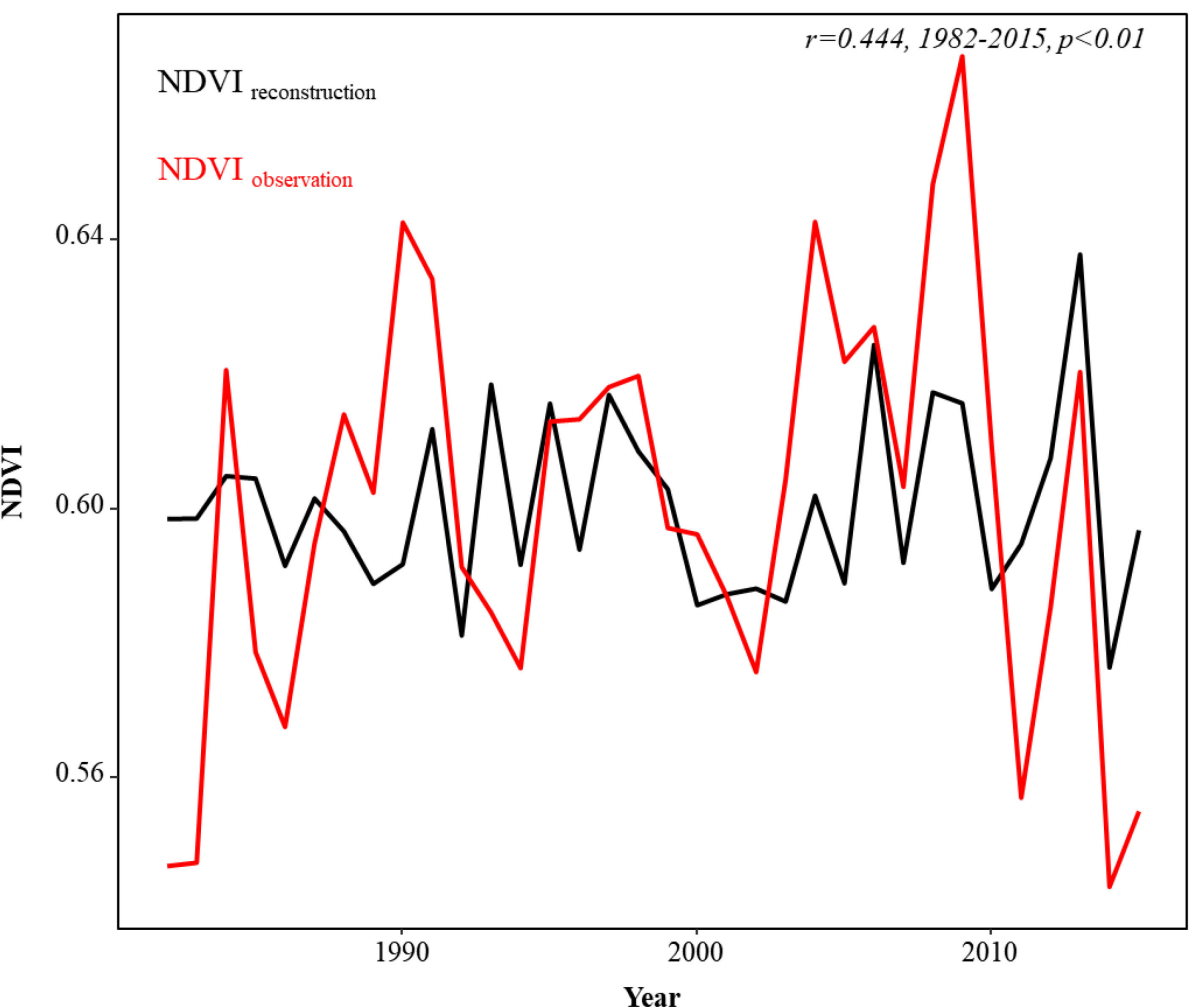
Comparison between reconstructed NDVI and measured NDVI.

To verify the reliability of the reconstructed model, we conducted a separate calibration test on the above two models. **Table 2** shows that the *F* value, *r*, ST, and ST1 were all statistically significant, and RE and CE were both positive, indicating that the regression models have been statistically validated (Cook and Kairiukstis, 1990; Peter, 2014). In addition, the comparison between the reconstructed and observed NDVI showed that the reconstructed NDVI captures the characteristics of NDVI changes well (*r* = 0.444, *p*< 0.01, **Figure 6**).

**Table 2:** Statistics of calibration and verification tests for summer NDVI reconstruction.

Calibration							Verification										
Period	*r*		*R* ^2^		*F*		Period		RE		CE		ST		ST_1_		
1982−1998	0.23		0.05		6.78		1999−2015		0.58		0.58		10+/7–*		10+/6–	
1999−2015	0.57		0.33		4.32		1982−1999		0.57		0.57		11+/6–*		11+/5–	
1982−2015	0.44		0.20		5.08											

“*” indicates the significance level of p< 0.05. Sign test (ST), One order different signal test (ST1), Reduction of error, RE), and Coefficient of efficiency (CE).

**Figure 7:**
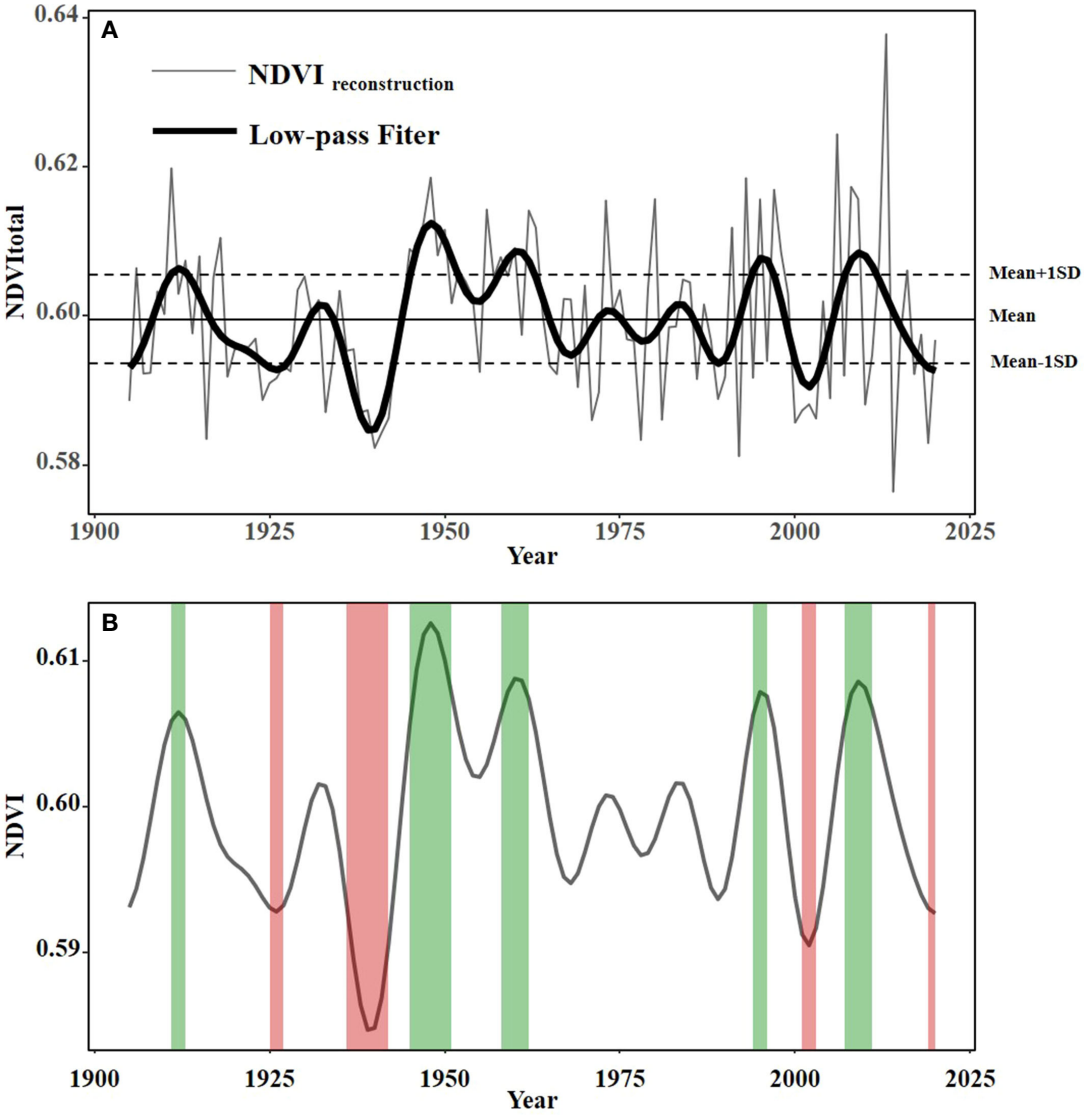
Change of vegetation: **(A)**The thin line represents the reconstructed summer NDVI, thick line represents the summer NDVI curve after 10 years of low-pass filtering, and the horizontal line indicates the mean and mean ± one standard deviation. **(B)** The thick lines represent 10 years of low flux data. The red shaded areas represent low cover intervals, while the green shaded areas represent high cover intervals.

Based on the above regression model, we reconstructed the summer NDVI from 1905 to 2020 in the mountainous and hilly areas in the central south of Shandong Province. In the past 115 years, summer NDVI varied from 0.576 to 0.638, with the mean value being 0.600 and the standard deviation (SD) being 0.006. It can be seen from **Figure 7A** that the interannual variation of vegetation cover in the mountainous and hilly area in the central-south Shandong Province is obvious since 1905. There were 23 years with high vegetation coverage, accounting for 20% of the whole series, while 15 years had low vegetation coverage, accounting for 13%. To study the decadal variation of vegetation cover in this area, we low-pass filtered the reconstructed NDVI for ten years and defined a year with higher than mean + 1SD as high vegetation cover while a year with lower than mean – 1SD as low vegetation cover. We found that there were four periods of low vegetation cover in this area, namely 1925–1927, 1936–1942, 2001–2003, and 2019–2020, and the duration was 3, 7, 3, and 2 years, respectively. There were five periods of high vegetation cover, namely 1911–1913, 1945–1951, 1958–1962, 1994–1996 and 2007–2011. The duration was 3, 7, 5, 3, and 5 years, respectively (**Figure 7**). The abnormal changes of regional vegetation cover may be related to some large-scale complex ecological and environmental events and human activities (Long et al., 2019). Vegetation changes caused by climatic factors and human activities will affect regional and global vegetation improvement or degradation (Gu et al., 2022), which in turn affects ground erosion resistance and surface runoff. And vegetation will be further affected when human activities intensify (Xu et al., 2021).

### 3.3 Impacts of climate change on vegetation coverage

Vegetation growth is affected by regional temperature variation and hydrological conditions (Guo et al., 2021). According to **Figure 5B**, we found that there was a significant correlation between summer NDVI and precipitation in the mountainous and hilly areas in the central south of Shandong Province, and the spring and summer seasonal precipitation significantly affected summer NDVI. The results showed that precipitation affected the summer vegetation growth in the study area.

Under the influence of the warm temperate monsoon climate, the precipitation in the mountainous and hilly areas in the central south of Shandong Province is concentrated in summer, and is obviously affected by ENSO events because of its location (Guo et al., 2017b). Because of this, the climate in the study area usually alternates between wet and dry. Research has shown that PDSI was high in the 1910s and 1950s-1970s in Shandong, which was at normal to slightly wet level, while PDSI showed a downward trend from the 1920s to the 1940s (Li et al., 2015). The lack of water inhibited the growth of plants and led to the reduction of vegetation coverage, while the abundant precipitation promoted the growth of plants, making the vegetation coverage significantly increase.

From the 1920s to the mid-1940s, the vegetation cover of the study area was always in a relatively low state (**Figure 7**). According to the History of Disasters in Modern Shandong, severe drought and famine occurred in Shandong during 1920–1921, 1928–1930, and 1942–1943, which not only reduced the vegetation coverage, but also threatened people’s lives and property (Wang, 2004). This is consistent with the results of Li et al. (2015). Drought affects photosynthesis directly and affects respiration and transpiration indirectly, causing plants to lack sufficient water and leading to partial dieback and reduced vegetation greenness dynamics (Yang et al., 2018). When soil water availability is low, plant photosynthesis at leaf level is affected by stomatal regulation and non-stomatal processes (Flexas and Medrano, 2002). Drought stress can reduce stomatal conductance and thus reduce CO_2_ assimilation in leaves (Zhu et al., 2021). In addition, long-term lack of adequate water supply would affect the biochemical process and limit the photosynthetic characteristics at leaf level by down-regulating the activity and content of Rubisco (DidionGency et al., 2022).

And according to “Chinese Meteorological Disaster Code: Shandong Volume”, it indicated that drought in Shandong Province was high probability, long duration, wide impact area, and great intensity. Typical droughts occurred in November 2010, March 2011, August 2014, and October 2015 in Shandong Province (Shen et al., 2020). When drought occurred, soil water availability decreased and plant physiological activities were inhibited due to water shortage, leading to plant dieback and decreased NDVI (Na et al., 2021). After drought events, the winter snow, spring, and summer rainfall timely supplemented the soil moisture, and the plants got sufficient water supply which made the summer NDVI rise. However, the drought in 2014 occurred in August, which directly and seriously affected summer NDVI, resulting in the lowest vegetation coverage in 2014 (**Figure 7**).

In addition, a prolonged drought combined with high winds could lead to forest fires (Silva et al., 2021). For example, several forest fires broke out in the remnant of Mount Tai at the junction of Jinan and Tai’an in Shandong Province in 2011 (http://www.chinanews.com/tp/hd/2011/04-19/33080.shtml), resulting in the destruction of the forest, and the vegetation coverage in the following years was lower than that before the fire (**Figure 7**).

### 3.4 Impacts of human activities on vegetation coverage

It can be seen from 3.3 that climate change (precipitation) is the dominant factor affecting vegetation coverage in the mountainous and hilly areas in the central south of Shandong Province, but the impacts of human activities on regional vegetation change cannot be ignored.

China’s climate has been warming since the 1950s, with surface air temperatures rising faster than the global average, which has significant impacts on water resources, agriculture, ecosystems, and human health (Sun et al., 2022). So, based on the correlation analysis between summer NDVI and climatic factors, we established a multiple linear regression model. The transfer function is as follows:


, $$NDVI_{climate}=0.0008060P_{C_{3-5}}+0.0003326P_{C_{6-8}}+0.5181292$$

(*n* = 34, 1982–2020 CE, *r* = 0.61, *R* ^2 = ^0.37, *R* ^2^_adj_ = 0.33, *p*< 0.01),

where ${\text{P}}_{{\text{C}}_{3-5}}$ is the average precipitation from March to May of the current year; and ${\text{P}}_{{\text{C}}_{6-8}}$ is the average precipitation from June to August of the current year.

Since *F* value, *r*, *p* are statistically significant, and *R* ^2^ is 37%, this regression model has been statistically verified (**Table 3**).

**Table 3:** NDVI_climate_ reconstruction equation statistical parameters.

r	R^2^	R^2^adj	F-text	
			F	p-value
0.61	0.37	0.33	2.69	< 0.01 **

“**” indicates the significance level of p< 0.01.

Then residual analysis was used to study the contribution rate of human activities to vegetation cover change in the study area since 1950.

**Figure 8:**
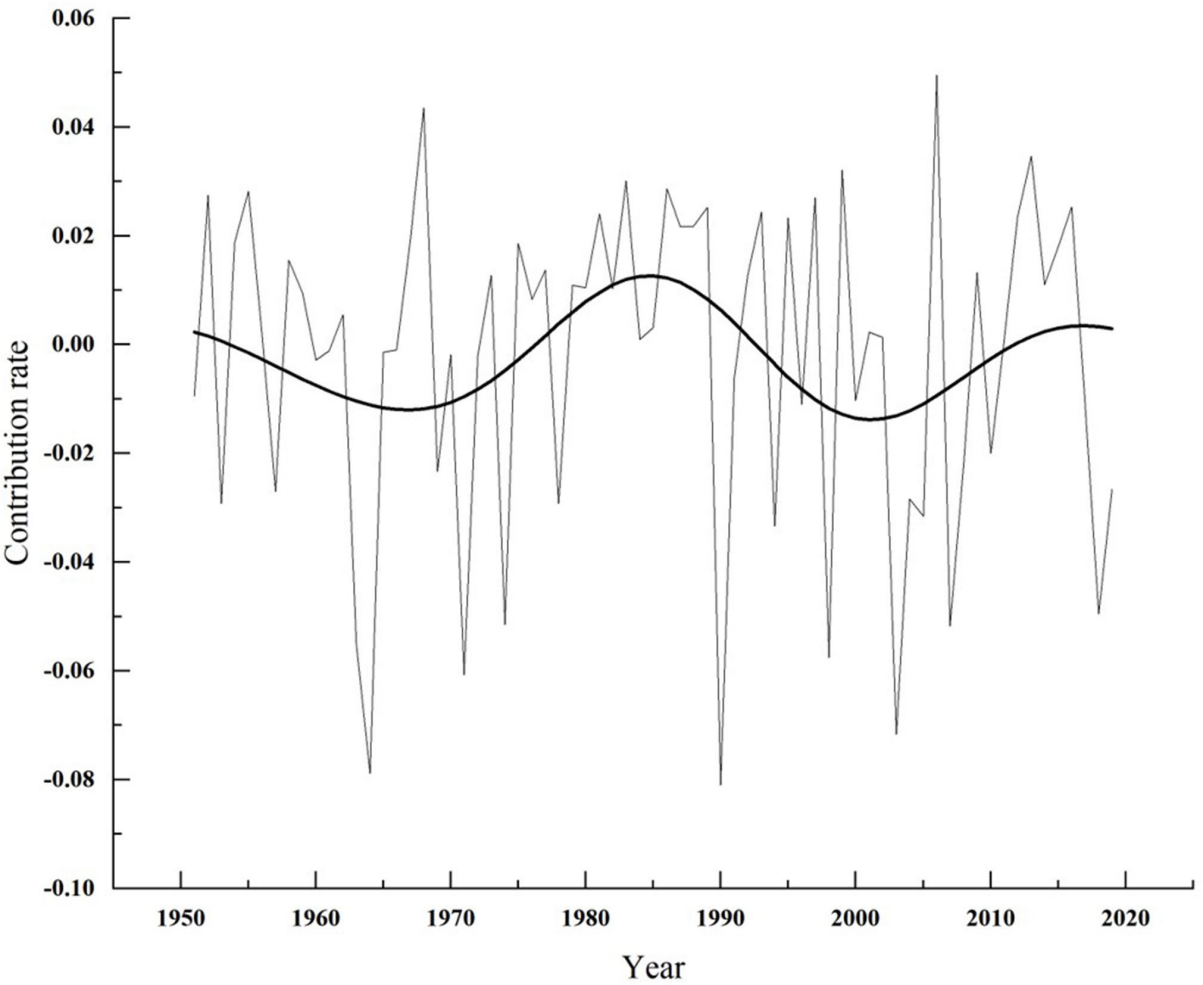
Contribution of human activities to dynamic change of vegetation cover in the mountainous and hilly region of central-south Shandong Province. The thin line represents the contribution of human activities to vegetation coverage changes, thick line represents the contribution curve after 20 years of low-pass filtering, and the horizontal line indicates the mean.

The results indicated that the contribution rate of human activities to vegetation cover change showed an increasing trend in fluctuation, and the positive and negative contribution to present regular continuous alternating change law (**Figure 8**). From **Figure 7B**, we knew the impact of human activities on vegetation coverage was in a relatively stable and positive state during 1979–1989. This is because the economic development and land use could be carried out in a planned way as a result of the Reform and Opening-up Policy in China (Li et al., 2018). Therefore, the vegetation coverage during this period also changed stably within a certain range (**Figure 8**).

The policy of Grain-for-Green, China’s largest program, was launched to alleviate land degradation by returning arable land on steep slopes to forest or grassland, while the Grain-for-Blue policy aimed to return farmland to water (Jia et al., 2014). After the policy of Grain-for-Green was implemented, grain subsidies and cash subsidies were given to the farmers since 2000. In 2007, the “Notice of The State Council on Improving the Policy of Grain-for-Green” stipulated that cash subsidies of 70 yuan per mu of returned farmland were given annually in the Yellow River Basin and northern regions (Zhang and Yu, 2012). During this period, the sown area of crops, sown area of grain, total grain output, and cultivated land area in the mountainous and hilly areas of central-south Shandong showed different degrees of decrease (Zhang et al., 2021). Therefore, the negative impact of human activities on vegetation cover has been reduced, and the vegetation coverage of the study area has shown a relatively increasing trend after the implementation of Grain for Green in Shandong Province since 2003 (**Figure 7**).

However, with the development of social economy, science, and technology, the urbanization level of the study area is constantly improving. The development level of rural local urbanization in the central-south of Shandong Province was at medium during 2011–2015, while the level improved to medium to high during 2016–2020 (Teng, 2022). Therefore, vegetation cover was affected, and it showed a downward trend after 2013 (**Figure 7**).

## 4 Conclusions

Based on the chronology of the tree-ring width of *P. orientalis*, NDVI data, and meteorological data, this study analyzed the impacts of climate change and human activities on vegetation coverage in the mountainous and hilly area in the central south of Shandong Province. The main conclusions are as follows:

1) In the past 115 years, the vegetation coverage in the study area showed obvious interannual and interdecadal variations. And there were 25 years with low vegetation coverage, while 23 years with high vegetation coverage. The study area experienced four periods of low vegetation coverage (1925–1927, 1926–1942, 2001–2003, and 2019–2020) and five periods of high vegetation coverage (1911–1913, 1945–1951, 1958–1962, 1994–1996 and 2007–2011).
2) Climate was the dominant factor affecting summer NDVI in the study area, especially the precipitation in spring (March–May) and summer (June–August), but the impacts of human activities on the vegetation coverage variation should not be ignored.
3) In this study, the effect of human activities to vegetation cover change showed an increasing trend in the fluctuation since 1950. With the development of social economy and technology, the policy system, ecological engineering, and rapid urbanization have improved or degraded the vegetation to different degrees.

Through this study, we were able to understand the relationship between climate change and vegetation cover, and the impact of human activities on vegetation cover in warm temperate regions of eastern China well. In addition, future studies should further analyze the importance degree of different types of human activities on vegetation cover.

## Data availability statement

The raw data supporting the conclusions of this article will be made available by the authors, without undue reservation.

## Author contributions

TY, HW, PZ, and RW proposed the study and designed the experiment. TY conducted field and laboratory measurements, and analyzed the data. HW, PZ, and RW secured fundings. YiZ, YaZ, and JD helped with laboratory measurements and data analysis. JD, XL, and WY helped with data analysis. TY wrote the manuscript that was intensively edited by all authors.

## Appendix

Running rbar (based on a 50-year window with a lag of 25 years) and running Expressed Population Signal (EPS).
